# Role of gut microbiota in infectious and inflammatory diseases

**DOI:** 10.3389/fmicb.2023.1098386

**Published:** 2023-03-27

**Authors:** Miriãn Ferrão Maciel-Fiuza, Guilherme Cerutti Muller, Daniel Marques Stuart Campos, Perpétua do Socorro Silva Costa, Juliano Peruzzo, Renan Rangel Bonamigo, Tiago Veit, Fernanda Sales Luiz Vianna

**Affiliations:** ^1^Postgraduate Program in Genetics and Molecular Biology, Universidade Federal Do Rio Grande Do Sul, Porto Alegre, Brazil; ^2^Instituto Nacional de Genética Médica Populacional, Porto Alegre, Brazil; ^3^Genomics Medicine Laboratory, Center of Experimental Research, Hospital de Clínicas de Porto Alegre, Porto Alegre, Brazil; ^4^Laboratory of Immunobiology and Immunogenetics, Department of Genetics, Universidade Federal Do Rio Grande Do Sul, Porto Alegre, Brazil; ^5^Department of Nursing, Universidade Federal do Maranhão, Imperatriz, Brazil; ^6^Dermatology Service of Hospital de Clínicas de Porto Alegre, Porto Alegre, Brazil; ^7^Postgraduate Program in Medicine, Medical Sciences, Universidade Federal Do Rio Grande Do Sul, Porto Alegre, Brazil; ^8^Postgraduate Program in Pathology, Universidade Federal De Ciências Da Saúde de Porto Alegre, Porto Alegre, Brazil; ^9^Department of Microbiology, Immunology and Parasitology, Institute of Basic Health Sciences, Universidade Federal do Rio Grande do Sul, Porto Alegre, Rio Grande do Sul, Brazil

**Keywords:** gut microbiota, microbiome, infectious diseases, inflammation, cytokines, immune modulation

## Abstract

Thousands of microorganisms compose the human gut microbiota, fighting pathogens in infectious diseases and inhibiting or inducing inflammation in different immunological contexts. The gut microbiome is a dynamic and complex ecosystem that helps in the proliferation, growth, and differentiation of epithelial and immune cells to maintain intestinal homeostasis. Disorders that cause alteration of this microbiota lead to an imbalance in the host’s immune regulation. Growing evidence supports that the gut microbial community is associated with the development and progression of different infectious and inflammatory diseases. Therefore, understanding the interaction between intestinal microbiota and the modulation of the host’s immune system is fundamental to understanding the mechanisms involved in different pathologies, as well as for the search of new treatments. Here we review the main gut bacteria capable of impacting the immune response in different pathologies and we discuss the mechanisms by which this interaction between the immune system and the microbiota can alter disease outcomes.

## 1. Introduction

**Figure 1:**
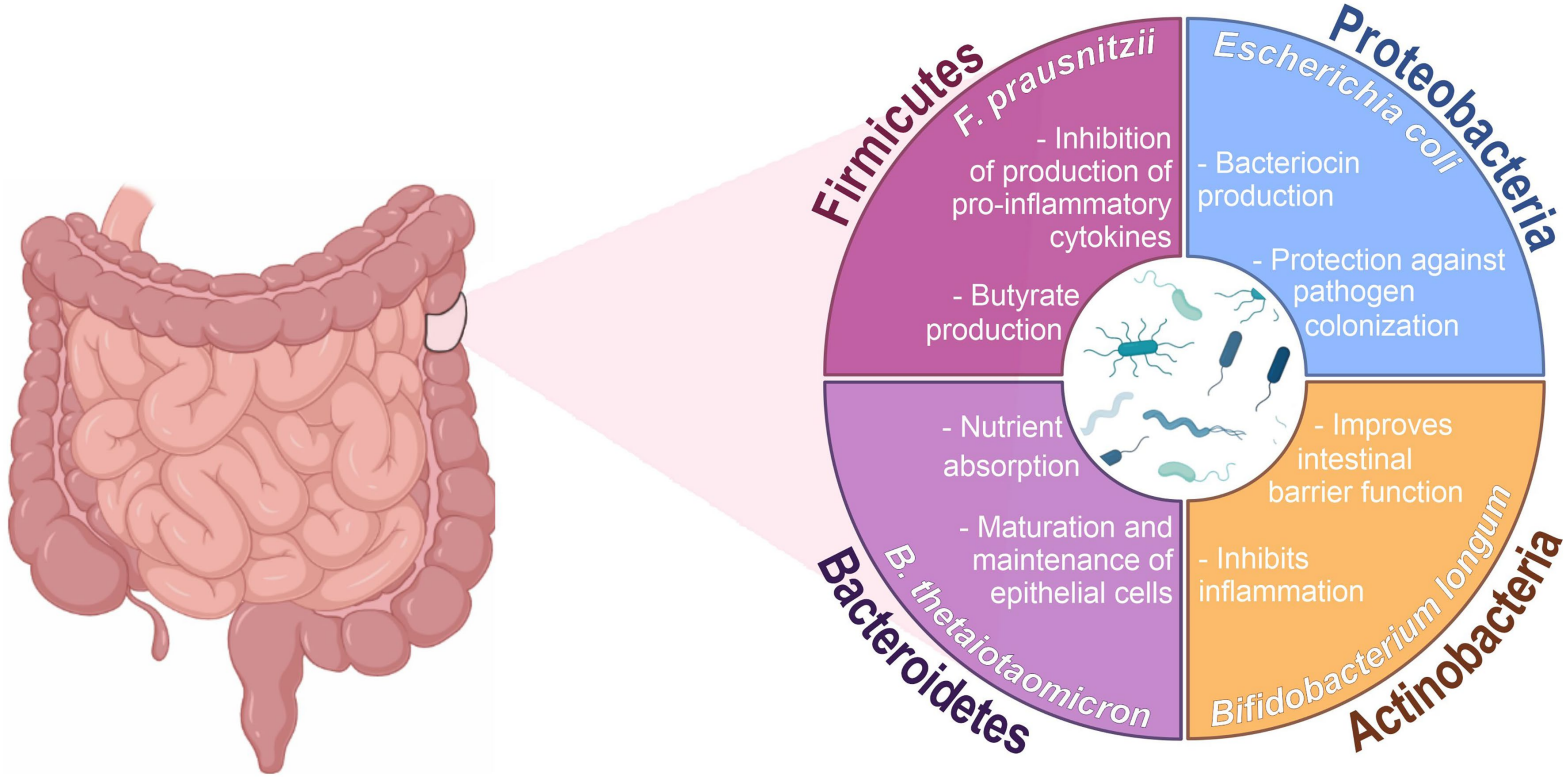
Main phyla and functions associated with the intestinal microbiota. Created with BioRender.com and coreldraw.com.

The human gut microbiota is a community of microorganisms that includes viruses, bacteria, archeas, fungi and protozoa, and the microbiome is the collective genomes of microorganisms, their metabolites, and proteins in a specific environment (Budden et al., 2019). In humans, the intestine harbors the greatest number of microorganisms and the greatest number of species in relation to other places in the body (Quigley, 2013). They consist of over 1,500 species, which colonize the digestive tract within minutes of birth, establishing a symbiotic or mutualistic relationship with epithelial and lymphoid tissue (Robles-alonso et al., 2013; Horta-Baas et al., 2017; Lourido et al., 2017; Budden et al., 2019; Mitev and Taleski, 2019). The intestinal microbiota is predominantly composed of bacteria, containing especially the phyla Firmicutes, Bacteroidetes, Actinobacteria and Proteobacteria (Figure 1), being the most microorganisms found in the colon (Acharya et al., 2017; El-Mowafy et al., 2021). These microorganisms produce a variety of metabolites from the anaerobic fermentation of exogenous dietary components and endogenous compounds generated by microorganisms and host. The generated metabolites, such as short-chain fatty acids (SCFAs), interact with host cells and influence immune responses (Hooper et al., 2002; Rooks and Garrett, 2016). Therefore, this is a dynamic and complex ecosystem that helps the proliferation, growth, and differentiation of epithelial cells to fight against infections and stimulate the immune system (Hara and Shanahan, 2007; Sultan et al., 2021a).

There is mounting evidence indicating that the gut microbiome plays an important role in modulating host physiology, with studies linking gut microbiome composition and functions to differential inflammatory, neurological, and even behavioral activities (Dinan and Cryan, 2017; Wipperman et al., 2021). The intestinal microbiota has several functions, including protection against pathogens by colonization of the mucosal surface and production of antimicrobial substances (Mills et al., 2019), aiding in digestion and metabolism (Rothschild et al., 2018), controlling the proliferation and differentiation of epithelial cells (Wiley et al., 2018), changing insulin resistance and affecting its secretion (Kelly C. J. et al., 2015; Kelly J. R. et al., 2015), influencing brain-intestinal communication and thus influencing host neurological functions (Zheng et al., 2019; Gomaa, 2020). Thus, disturbances in the intestinal microbial population can result in an imbalance of the homeostasis, promoting the development of pathologies (Mori et al., 2021). Several intrinsic factors can influence the composition and function of the gut microbiota, such as birth form, age, host genetics and innate and adaptive immunity. Extrinsic factors such as diet, lifestyle, geographic region, presence of allergens or pathogens and antibiotic therapy can also determine the type of microorganism found (Rodrı et al., 2015; Martinez and Taddei, 2018; Hasan and Yang, 2019). Dysbiosis is a term used to describe a quantitative and/or qualitative change in the composition of the microbiota (Passos and Moraes-Filho, 2017). Dysbiosis can be caused by many of daily activities, such as dietary patterns, hygiene habits, physical activity, and medication use (Mitev and Taleski, 2019). When there is a dysbiotic state, the functioning of the microbiota is affected and can induce a disease state (Schwiertz, 2016; Lee and Kim, 2017). In this review, we provide an overview of the current understanding of the role of the gut microbiota in the regulation of the immune system and the modulation of serum cytokines in the most common and/or most studied autoimmune and inflammatory diseases, and in viral and mycobacterial infections.

## 2. Microbial metabolites and immune system

The human gastrointestinal tract is the main site of interactions between microorganisms and the host’s immune system. In this interaction, the microbiota contributes to the physiological functions of the host while the host provides nutrition and habitat (Caricilli, 2014). The gut microbiota is essential not only for the degradation and fermentation of feed, but also for defense against pathogens, either by competing for nutrients and adhesion sites, or by secreting antimicrobial peptides (Moens and Veldhoen, 2012; Kamada et al., 2013; Takiishi et al., 2017). Experiments conducted in germ-free animals (GF) have demonstrated that colonization of the microbiota early in life is necessary for the proper development of the immunity. In the lack of gut microbiota, the immune system of the intestinal mucosa is underdeveloped, with, for example, reduced number of functional regulatory CD4+ CD25+ T cells, resulting in a reduced capacity to fight pathogenic bacteria (Hara and Shanahan, 2007; Sommer and Bäckhed, 2013; Takiishi et al., 2017). In addition, the balance between proinflammatory interleukin (IL)-17-producing effector T helper (Th17) cells and Forkhead box P3 (Foxp3^+^) regulatory T (Tregs) cells in the gut requires signals from gut bacteria, and those signals are dependent on gut microbiota composition (Ivanov and Littman, 2010). For instance, GF animals colonized with *Bacteroides fragilis* had the balance between Th1 and Th2 cells restored, thanks to the production of polysaccharide A (Mazmanian et al., 2005). Polysaccharide A is a bacterial product that influences T cell activation through interaction with Toll-like Receptor 2 (TLR2). It inhibits Th17 differentiation and favors Treg activity, thus favoring immune tolerance (Round et al., 2011). Resident bacteria, especially Clostridia-related species, have been associated with development of Th cells and induction of Treg cells (Gaboriau-Routhiau et al., 2009; Atarashi et al., 2011; D’Amelio and Sassi, 2018).

The intestinal microbiota produces a diverse repertoire of metabolites from food by modifying host products and by *de novo* synthesis. Among them, short-chain fatty acids (SCFAs) are the most described in the regulation of the immune system (D’Amelio and Sassi, 2018). SCFAs result from fiber fermentation in the colon and include acetic acid, butyric acid, and propionic acid, which cross the intestinal epithelium and interact with host cells, influencing immune responses (Takiishi et al., 2017). In addition to their metabolic functions, these substrates have several regulatory functions. SCFAs are inhibitors of histone deacetylases (HDACs) and ligands for G protein-coupled receptors (GCPRs), also called free fatty acid receptors (FFAR). SCFA-guided inhibition of HDACs tends to promote a tolerogenic and anti-inflammatory cell phenotype that is essential for maintaining immune homeostasis (Rooks and Garrett, 2016).

Studies with exposure of peripheral blood mononuclear cells (PBMCs) and neutrophils to SCFAs showed inhibition in the production of the pro-inflammatory cytokine tumor necrosis factor (TNF) and in the activation of nuclear factor-κB (NF-κB; Usami et al., 2008; Rooks and Garrett, 2016). SCFAs also influence peripheral T cells, especially regulatory T cells, through HDAC inhibition. Tao et al. (2007) reported that inhibition of HDAC9 increased the expression of Foxp3^+^ and number of Treg cells, improving suppressor function of Foxp3^+^ Treg cells under homeostatic conditions and amplified attenuation of Treg cell-mediated colitis in mice. Some SCFAs such as butyrate and propionate also modulate antigen presentation by inhibiting dendritic cell development through inhibiting HDACs (Bernard et al., 2002; Wang et al., 2008; Singh et al., 2010; Liu L. et al., 2012) and interacting with FFAR (Singh et al., 2010; Arpaia et al., 2013; D’Amelio and Sassi, 2018).

Furthermore, by regulating the activity of hypoxia-inducible factor (HIF), butyrate and propionate are associated with the maintenance of intestinal homeostasis (Kelly C. J. et al., 2015). HIF is the main regulator of oxygen homeostasis in response to hypoxia (Brahimi-Horn and Pouysségur, 2007; Rocha, 2007). It is a transcription factor formed by a heterodimeric protein, composed of α and β subunits. The β subunit, also called the aryl hydrocarbon receptor nuclear translocator (ARNT), is not influenced by oxygen and is stably expressed. The α subunit, composed of three subunits (HIF-1α, HIF-2α, HIF-3α), is directly regulated by the presence of oxygen (EMA et al., 1997; Muz et al., 2009; Dengler et al., 2014). In a situation of tissue normoxia, HIF-1α is continuously synthesized and degraded through the 26S proteasome system. In contrast, under hypoxic conditions, HIF-1α stabilizes and bound to HIF-1β, initiates transcription of its target genes. In the intestine, these target genes are basally regulated to maintain the epithelial barrier and include genes crucial for cellular energetics (Glover et al., 2016), barrier function (Furuta et al., 2001), mucin production (Louis et al., 2006), microbial defense (Kelly et al., 2013), and xenobiotic clearance (Wartenberg et al., 2003). Therefore, HIF-1α stabilization maintains the structure of the epithelial barrier (van Itallie and Anderson, 2014), stimulates CD4+ T cells and IL-22 production (Yang et al., 2020) and, increases the expression of MUC2, MUC3 and intestinal trefoil factor (ITF), which is essential for the epithelial restoration of the colon (Louis et al., 2006; Dilly et al., 2016; Glover et al., 2016; Ma S. et al., 2022). Thus, SCFAs play an important role in regulating the host–microbe interaction, modulating the HIF, which directly influences this crosstalk.

In addition to SCFAs, other metabolites produced by the gut microbiota have important immunomodulatory functions, such as indole derivatives, which are derived from tryptophan, and polyamines, originated from dietary arginine. Indole derivatives promote the integrity of the enteric epithelium and the defense against microorganisms, inducing the multiplication of intestinal goblet cells, and the secretion of antimicrobial peptides, and mucins (D’Amelio and Sassi, 2018). Tryptophan derivatives also promote the differentiation and function of anti-inflammatory macrophages, Treg cells and IL-22 producing innate lymphoid cells 3 (ILC3). IL-22 acts in the maintenance of intestinal epithelial cells (IECs), regulates the equilibrium of the commensal microbiota and protects against infection by *Citrobacter rodentium* (Liang et al., 2006; Su et al., 2022). In mice, ILC3s induce fucosylation, which is an important glycosylation mechanism in IECs. This induction may be dependent on commensal bacteria, using IL-22, and independent of these bacteria, requiring lymphotoxin. The absence of intestinal fucosylation leads to increased susceptibility to *Salmonella typhimurium* infection. Therefore, ILC3s play an important role in modulating the intestinal microenvironment through the regulation of epithelial glycosylation, protecting against infection by pathogenic bacteria (Goto et al., 2014). Polyamines, such as putrescine, are found in many cells and play a role in gene transcription, translation, proliferation, and cell death. Polyamines are essential for host cell functions; for example, intestinal epithelial cells depend on these molecules to maintain high proliferation rates. They assist the development and maintenance of the intestinal epithelium and the inhabiting immune cells (Rooks and Garrett, 2016; D’Amelio and Sassi, 2018).

Most evidence suggests that intestinal microbiota metabolites and antigens can influence the immune system. Therefore, dysbiosis, characterized by alterations in the microbiome resulting in an imbalance in the microbiota, can contribute to the development of some immunological and inflammatory pathologies, both at the intestinal level, such as the well-documented Inflammatory Bowel Disease (IBD; Lane et al., 2017), and in other regions of the body, such as in rheumatoid arthritis (RA; Maeda et al., 2016). Indeed, many organs distant from the intestine, such as the skin, brain, and lungs, which are not in direct contact with the intestinal microbiota, can be affected by dysbiosis and its repercussions in the immune point of view. This suggests that the gut microbiota actually has the capacity to interact with the immune system in a systemic manner. For this, the gut microbiota needs to send microbial signals that are transmitted through the intestinal epithelium. These signals can be structural components of the bacteria or the metabolites themselves produced by the gut microbiota that can diffuse through the circulation and directly affect distant organs or by signaling nerves or hormones in the gut (Schroeder and Bäckhed, 2016; D’Amelio and Sassi, 2018).

## 3. Gut microbiota and cytokine modulation

The gut microbiota is mainly composed of the phyla Bacteroidetes and Firmicutes, which comprise approximately 90% of the microbial population in humans (Eckburg et al., 2005; Qin et al., 2010). Bacteroidetes vary in relative abundance among individuals, but they normally make up half of the gut microbiome. The members of this phylum reside especially in the distal intestine, where they function in the fermentation of indigestible carbohydrates. The predominant genera of Bacteroidetes in the human gastrointestinal tract are *Bacteroides*, *Prevotella* and *Porphyromonas* (Qin et al., 2010; Huttenhower et al., 2012).

**Figure 2:**
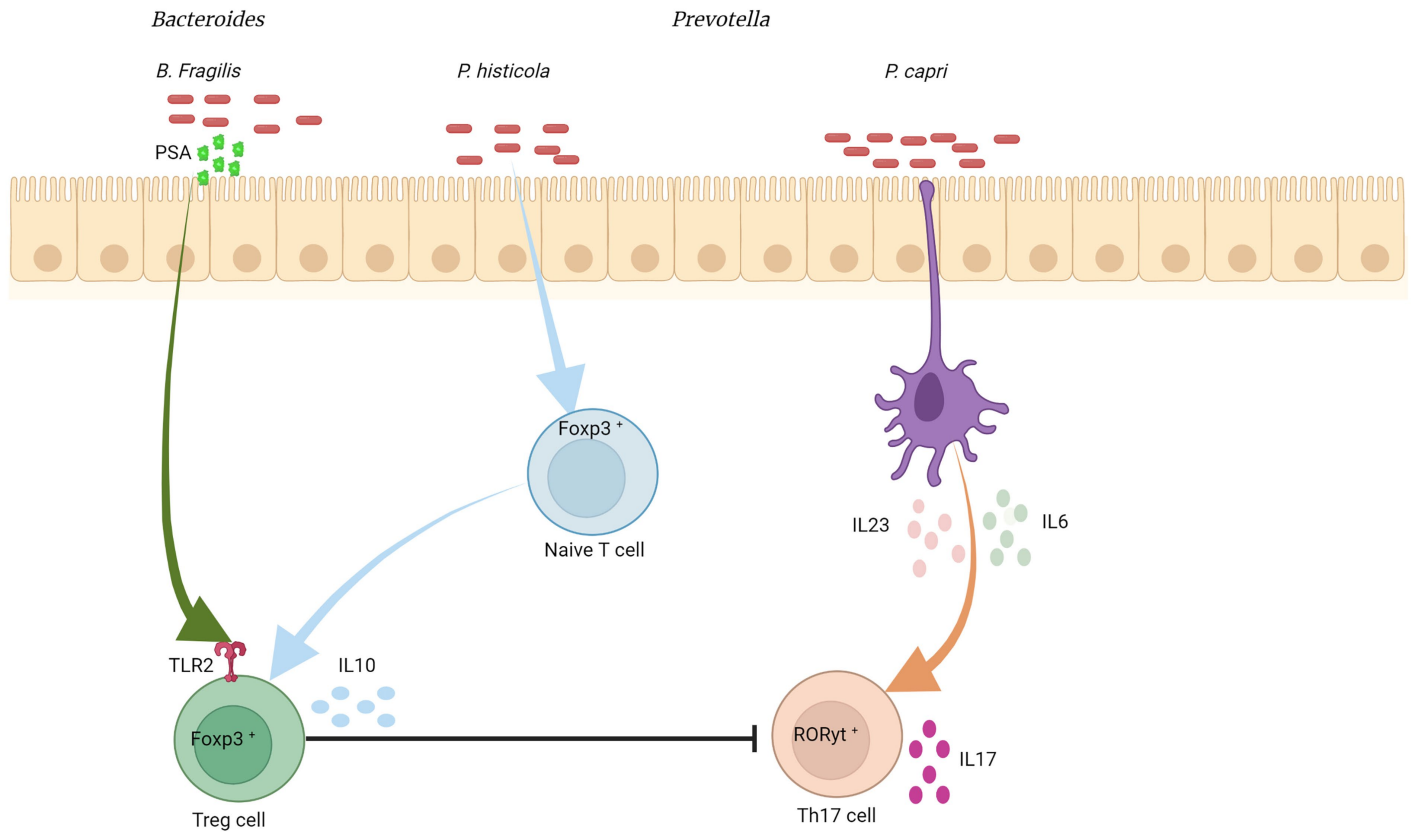
Bacteroides/Prevotella colonization and cytokine modulation. *Bacteroides fragilis* was associated with the induction of an anti-inflammatory response, inducing the differentiation of CD4+ T cells into Treg cells, which produce IL-10 and suppress Th17. This differentiation into Treg is mediated by TLR2 (from CD4+ cells), which recognizes polysaccharide A (from the bacterial outer membrane), activating a signaling cascade. *Prevotella copri* stimulates dendritic cells to express high levels of IL-6 and IL-23, which may increase the number of intestinal Th17 cells. *P. histicola* suppresses serum levels of pro-inflammatory cytokines such as IL-2, IL-17, and TNF-α, by increasing Treg cells in the gut and reducing Th17 cell responses. Created with Biorender.com.

Pro- and anti-inflammatory cytokines play an important role in regulating the host’s immune response to the intestinal bacteria’s own compositional variations and, therefore, in maintaining intestinal balance. For example, **interleukin-10** (IL-10) production by Tregs is essential in maintaining intestinal homeostasis, as it prevents excessive inflammation. *Lactobacillus rhamnosus* and *Lactobacillus reuteri* have been shown to induce IL-10 production by Tregs (Liu Y. et al., 2012; Jang et al., 2019). **Interleukin-17A** (IL-17A), produced by Th17 cells, is an important mediator of innate and adaptive immune response, but can also contribute to inflammation and tissue damage. Some members of the *Bacteroides*, such as *B. fragilis*, have been shown to induce Treg differentiation from CD4+ T cells, and thus decrease IL-17A production (Round et al., 2011). On the other hand, *Prevotella* spp. have been associated with an increase in IL-17A production in CD4+ T cells (Figure 2; Maeda et al., 2016). **Interleukin-22** (IL-22), also produced by Th17 cells, and innate lymphoid type 3 (ILC3) cells and is involved in defense against extracellular pathogens. IL-22 production is also associated with epithelial regeneration and repair. *Akkermansia muciniphila*, a common human gut bacterium, has been shown to induce IL-22 production by ILC3 cells (Bachmann et al., 2022; Zheng et al., 2023). **Interferon gamma** (IFN-γ) production is a typical response of Th1 cells and is associated with protective immunity against intracellular pathogens. Supplementation with different species of *Lactobacillus* increased IFN-γ production by T lymphocytes, macrophages, and dendritic cells (Won et al., 2011; Dimitrijevic et al., 2014; Kim et al., 2015; Saliganti et al., 2015; Yang et al., 2015). **Interleukin-12** (IL-12), which is a key factor in the polarization of CD4+ T cells to the Th1 phenotype, can also be modulated through the gut microbiota. Several strains of *Lactobacillus* also have been associated with an increase in IL-12 production (Christensen et al., 2002; Lee et al., 2013, 2015; Kim et al., 2015). Therefore, the intestinal microbiota can modulate and be modulated by cytokines produced in the intestine. Directly and indirectly influencing host immune responses in states of health and disease. However, the impact of the different compositions of the intestinal microbiota on the modulation of cytokine production and consequent inflammatory response needs to be better elucidated.

## 4. The gut microbiota and autoimmune/inflammatory diseases

The immune system is a collection of cells, tissues and organs that work together in complex ways to protect the body from invaders. It is composed of several blood cells such as dendritic cells (DCs), T cells and B cells, lymphoid organs such as bone marrow, and lymph nodes, and molecules as antibodies, complement and cytokines. The function of the immune system is to eliminate infectious microorganisms and cancer cells, and to aid repair tissue after injury, thus contributing to the maintenance and reestablishment of homeostasis. In autoimmune diseases, the balance between pathogen recognition and self-attack prevention is compromised. As a result, control of inflammation is lost and continued activation of the immune system occurs even in the absence of infection (Wahren-Herlenius and Dörner, 2013; Kuwabara et al., 2017).

Autoimmune and inflammatory diseases are characterized by dysregulated immune response, with production in abnormal amounts of autoantibody-producing B cells, autoreactive T cells, and augmented production of pro-inflammatory cytokines (Raphael et al., 2015; Kamali et al., 2019). Genetic and environmental factors including geographic location, immunological disorders and viral infections favor the development of autoimmune diseases. Furthermore, dysbiosis of the intestinal microbiota has been associated with these pathologies through several mechanisms, which can impact the regulation of the human immune system (Table 1). For example, molecular mimicry (when self-antigens and foreign antigens share similar sequences or structures) impacts on the permeability of the intestinal mucosa and may be associated with initiation and amplification of disease progression. While certain microbiota compositions could prevent autoimmunity in genetically susceptible individuals, disturbances or alterations in this composition may trigger the autoimmune process (Kawajiri and Fujii-Kuriyama, 2017; Xu et al., 2019).

**Table 1:** Autoimmune diseases and alteration of the gut microbiota composition.

Disease	Species	Increase microbiota species	Depletion microbiota species
SLE	Human^1^, Mouse^2^, Human and Mouse^3^	Genus: *Bacteroides* (Wei et al., 2019)^1.^, *Rhodococcus*, *Eggerthella*, *Klebsiella*, *Prevotella*, *Eubacterium*, and *Flavonifractor* (He et al., 2016)^1^	Phylum: *Firmicutes* and *Bacteroidetes* (He et al., 2016; HEVIA et al., 2014; Rodríguez-Carrio et al., 2017;^1^ van der Meulen et al., 2019)^1^
RA	Human^1^, Mouse^2^, Human and Mouse^3^	*Prevotella copri* (Scher et al., 2013; Maeda et al., 2016)^3,1^	Genus:*Bacteroides* (Scher et al., 2013; MAEDA et al., 2016)^3,1^
		*Lactobacillus salivarius* (Zhang et al., 2015)^1^	*Haemophilus* spp. (Zhang et al., 2015)^1^ and Genus *Faecalibacterium* (Chen et al., 2016)^3^
		*Collinsella aerofaciens* and *Eggerthella lenta* (Chen et al., 2016)^3^	
IBD	Human^1^, Mouse^2^, Human and Mouse^3^	Phylum *Proteobacteria*, Family *Enterobacteriaceae*, *Bilophila* and certain members of phylum *Bacteroidetes* (Zhou et al., 2018)^1^	*Akkermansia muciniphila* (PNG et al., 2010)^1^
			*Bifidobacterium* spp. (Joossens et al., 2011; Andoh et al., 2012)^1^, *Lactobacillus* spp. (OTT et al., 2004)^1^, and *F. prausnitzii* (Sokol et al., 2009; Joossens et al., 2011; Andoh et al., 2012)^1^

^1^Human model. ^2^Mouse model. ^3^Human e Mouse model. SLE, systemic lupus erythematous; RA, rheumatoid arthritis; IDB, inflammatory bowel disease.

### 4.1. The gut microbiota and inflammatory bowel disease

Inflammatory bowel disease (IBD) is a group of complex multifactorial inflammatory diseases that affect the gastrointestinal tract (Xavier and Podolsky, 2007; Jergens et al., 2021). It comprises two main classes: ulcerative colitis (UC) and Crohn’s disease (CD), which have different clinical, endoscopic, immunological, and histopathological characteristics (Jergens et al., 2021; Sultan et al., 2021a). UC is the most common form of IBD, which affects more than 5 million individuals worldwide (Alatab et al., 2020; Yang et al., 2021). Its inflammation is limited to the mucous layer, causing superficial damage restricted to the wall of the rectum and colon (Kobayashi et al., 2020; Arukha et al., 2021). CD is characterized by irregular transmural inflammation that extends through the intestinal wall into the serous layer, and it affects mainly the terminal ileum, but it can affect any part of the gastrointestinal tract (Arukha et al., 2021; Jergens et al., 2021).

Both diseases are characterized by an imbalance between anti and proinflammatory signals and the displacement of leukocytes to the intestinal epithelium. However, the T cells populations involved in the immune responses seem to be different depending on the disease, which may explain the different phenotypes observed in clinical practice (Ramos and Papadakis, 2019). UC is thought to occur due to an imbalance of intestinal immunity related to Th2 cytokines, while CD is associate to a Th1 and Th17 cytokine profile (Heller et al., 2005). In CD, differentiation into Th1 and Th17 occurs by induction of cytokines IL-12, IL-18, IL-23 and transforming growth factor beta (TGFβ) produced by macrophages and other antigen-presenting cells (APCs; Ramos and Papadakis, 2019). In UC, increased secretion of IL-5, which is Th2 specific, is related to more effective activation of B cells and stimulation of immune responses when compared to the Th1 response observed in CD (Ramos and Papadakis, 2019). Although the exact mechanism of causing IBD remains unknown, it is broadly accepted that the pathogenesis of the disease involves the interaction of genetic susceptibility and environmental factors in the microbiome, which, through an impaired intestinal epithelium, will lead to excessive immune activation, responsible for the clinical observed in patients (Ma et al., 2019; Ramos and Papadakis, 2019; Sultan et al., 2021a). Thus, genetically susceptible subjects are thought to produce a disordered immune response to their gut microbiota, leading to chronic inflammation and repetitive damage to the intestinal mucosa (Sartor, 2008; Jergens et al., 2021).

IBD is one of the diseases most associated with dysbiosis of the gut microbiota (Xu et al., 2019). Patients with IBD show loss of microbial diversity and stability and an increase in Proteobacteria and certain members of Bacteroidetes (Bautzova et al., 2018; Xu et al., 2019; Barbara et al., 2021). Among the components of Proteobacteria, adherent/adhesive *Escherichia coli* strains have been associated with IBD (Darfeuille-Michaud et al., 2004; Sokol et al., 2006). Adherent invasive *E. coli* was associated with CD, while diffusely adherent *E. coli* was associated with UC (Chloé Mirsepasi-Lauridsen et al., 2019). Adherent invasive *E. coli* is able to adhere to the intestinal mucosa, invade and replicate within IECs, translocate through the intestinal barrier and move to deeper tissues (Darfeuille-Michaud, 2002; Barnich et al., 2007). Furthermore, adherent invasive *E. coli* survives within macrophages, induces TNF-α production, and promotes granulomatous inflammatory response (Barnich et al., 2007; Meconi et al., 2007). Diffusely adherent *E. coli*, on the other hand, is able to adhere to the colonic mucosa and induce inflammatory responses characterized by induction of cytokine secretion, including IL-8, TNF-α and IL-1β and by promoting increased intestinal permeability (Servin, 2005; le Bouguénec and Servin, 2006). These data suggest that *E. coli* strains may play a key role in the pathogenesis of IBDs (Chloé Mirsepasi-Lauridsen et al., 2019).

**Figure 3:**
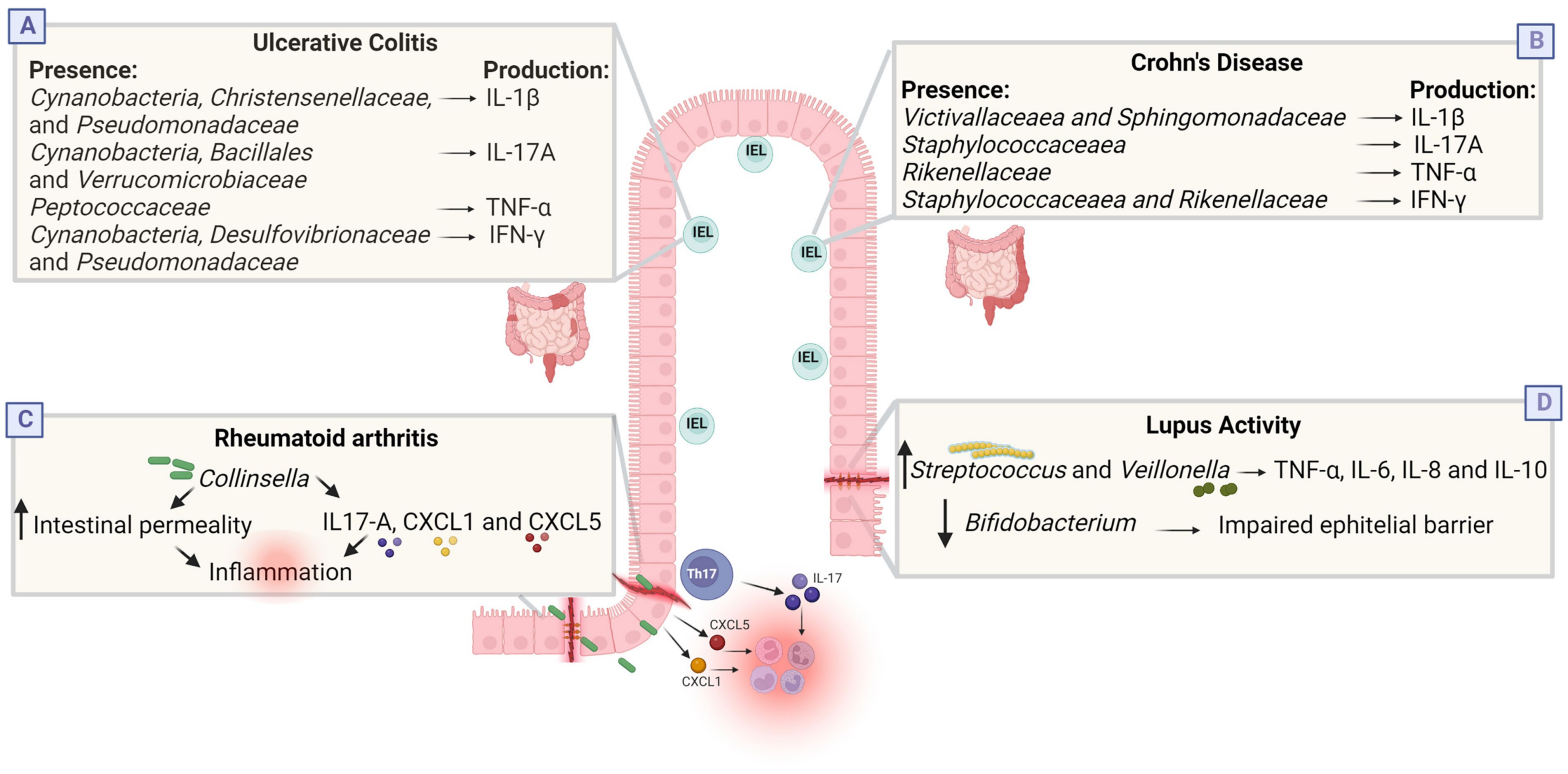
Dysbiosis in inflammatory and infectious diseases. **(A,B)** Cytokines produced by intestinal intraepithelial lymphocytes (IEL) correlate with the relative abundance of some bacterial taxa in IBD. In the UC group, there was positive correlation between the abundances of *Cynanobacteria*, *Christensenellaceae*, and *Pseudomonadaceae* and IL-1β; between *Cynanobacteria*, *Bacillales*, and *Verrucomicrobiaceae* and IL-17A; between *Peptococcaceae* and TNF-α; and between *Cynanobacteria*, *Desulfovibrionaceae*, and *Pseudomonadaceae* and IFN-γ. In the CD group, there was positive correlation between the abundances of *Victivallaceaea* and *Sphingomonadaceae* and IL-1β; between *Staphylococcaceaea* and IL-17A; between *Rikenellaceae* and TNF-α; and between *Staphylococcaceaea* and *Rikenellaceae* and IFN-γ. **(C)** Culture with *Collinsella* reduces the expression of junction proteins, increasing intestinal permeability, and influences the secretion of IL-17A, CXCL1 and CXL5, which can trigger neutrophil recruitment and NFkb activation, possibly increasing pro-inflammatory conditions in RA. **(D)** Lupus activity was positively associated with the genera *Streptococcus*, *Campylobacter* and *Veillonella*, and negatively correlated with *Bifidobacterium*. *Streptococcus* combined with *Veillonella* enhance the TNF-α, IL-8, IL-6, and IL-10 response while *Bifidobacterium* is associated with improved gastrointestinal barrier function and suppression of pro-inflammatory cytokines. Together, these changes possibly induce an inflammatory state. Created with Biorender.com.

Unlike *E. coli*, *Akkermansia muciniphila* has been shown usually reduced in the intestine of patients with IBD, resulting in an increase in the overall population of mucosal bacteria (Png et al., 2010; Barbara et al., 2021). IBD patients also have a lower abundance of *Lactobacillus* spp. (Ott et al., 2004), *Bifidobacterium* spp. (Joossens et al., 2011; Andoh et al., 2012), and *F. prausnitzii* (Sokol et al., 2009; Joossens et al., 2011; Andoh et al., 2012) resulting in reduced SCFAs concentrations when compared to healthy individuals (Huda-Faujan et al., 1967; Sultan et al., 2021a). Through its ability to produce butyrate *F. prausnitzii* performs anti-inflammatory activity. Butyrate improves intestinal barrier function and regulates the balance between Treg and Th17 cells (Zhou et al., 2018). Furthermore, Regner et al. reported that intestinal intraepithelial lymphocytes (IELs) and cytokines produced by these cells correlated with the relative abundance of various bacterial taxa. IELs from individuals with UC and CD produce different cytokines when compared to controls. In UC, IELs secrete increased amounts of IL-1β, while in CD there is increased secretion of IL-17A, IFN-γ and TNF-α (Figure 3; Regner et al., 2018). IELs are T cells that are in close contact with gut bacteria and can be influenced by differences in gut microbiota components (Regner et al., 2018; Xu et al., 2019). Together, these data suggest that dysbiosis in IBD patients could lead to the loss or impairment of microbial functions necessary to maintain intestinal epithelial barrier integrity, possibly causing increased inflammatory responses and spread of pathogens to intestinal tissues. However, is still unknown if these changes are a cause or consequence of IBD (Barbara et al., 2021).

### 4.2. The gut microbiota and rheumatoid arthritis

Rheumatoid arthritis (RA) is a systemic autoimmune disease characterized by a constant immune response that results to chronic inflammation and destruction of cartilage and bones. It is a serious chronic disease that affects about 1% of the world’s population, being more common in women than in men (Horta-Baas et al., 2017; Li Y. et al., 2019; Bergot et al., 2020). The mechanisms involved in the etiopathogenesis of the disease are complex and encompass both innate and adaptive immune responses, involving APCs, generation of autoreactive T cells and production of autoantibodies, such as rheumatoid factor (Horta-Baas et al., 2017; Pan et al., 2020). In addition, an association of the intestinal microbiome with the development and progression of RA has been demonstrated (Mangalam et al., 2021). An altered gut microbiota has been associated with loss of tolerance to autoantigens and in the increase of inflammatory episodes that cause damage to the joints (Xu et al., 2019). Furthermore, patients with RA have a reduction in the diversity of the gut microbiota when compared to controls and this is correlated with duration of illness and levels of autoantibodies produced (Chen et al., 2016; Xu et al., 2019).

Some individuals with early rheumatoid arthritis (who have not treated with antirheumatic drugs) have a greater relative abundance of *Prevotella copri* and a decrease in the number of *Bacteroides* in the gut (Scher et al., 2013; Maeda et al., 2016; Schwiertz, 2016; Maeda and Takeda, 2017). A study in China identified that RA patients had an increase in the abundance of *Lactobacillus salivarius* in the gut, teeth, and saliva. In contrast, *Haemophilus* spp. were decreased in these patients at all sites evaluated (Zhang et al., 2015). In another study, patients with RA also had decreased intestinal microbial diversity, which correlated with antibody production and illness duration. RA patients showed an increase in the relative abundance of *Collinsella aerofaciens* and *Eggerthella lenta* and a decrease in *Faecalibacterium* (Chen et al., 2016; Maeda and Takeda, 2019). In *in vitro* experiments, the genus *Collinsella* increased intestinal permeability and induced IL-17A expression, suggesting that the expansion of the microorganisms of this genus increases proinflammatory conditions, thus being an arthritogenic candidate in the human intestine (Figure 3; Nielen et al., 2004; Xu et al., 2012; Chen et al., 2016; Jiao et al., 2020). The reduction in the abundance of *Faecalibacterium* may be associated with a reduction in the production of butyrate, a final metabolite of fiber breakdown that presents an anti-inflammatory property, maintaining the integrity of the intestinal epithelium (Kim et al., 2016; Zhong et al., 2018; Gioia et al., 2020).

Intestinal microbiota involvement appears to vary in different subsets of RA patients (Chiang et al., 2019). However, despite the discrepancies found in different studies, *P. copri*, *L. salivarius*, and *Collinsella* are predominant in recent early RA and may be associated with its pathogenesis. Differences in patient characteristics, such as genetic background, environmental exposures and different treatment regimens may explain the variety of candidate arthritogenic bacteria (Chiang et al., 2019; Maeda and Takeda, 2019).

### 4.3. The gut microbiota and systemic lupus erythematosus

Systemic lupus erythematosus (SLE) is a chronic autoimmune disease that affects multiple structures in the body (Luo et al., 2018; Muhammad Yusoff et al., 2020). It is characterized by persistent inflammation in organs and presents several clinical manifestations, including skin rash, neurological disorders, glomerulonephritis, and severe vasculitis (Xu et al., 2019; Gu et al., 2020). SLE is more frequent in women, being triggered by the interaction between different factors, such as genetic predisposition, hormonal changes, environmental factors, and epigenetics. Despite this, the exact etiology and pathogenesis of the disease remain unknown (Luo et al., 2018; Guo et al., 2020).

Several immunological alterations have been reported in human and animal models of SLE, including autoreactive B and T cells, abnormal levels of pro-inflammatory cytokines and impaired immune complex clearance. This loss of self-tolerance plays a fundamental role in the occurrence and development of the disease (Luo et al., 2018; Muhammad Yusoff et al., 2020). Ineffective elimination and/or excessive formation of neutrophil extracellular traps (NETs), characterized by fibrous networks made up of nuclear and granular components that protrude from the membrane of activated neutrophils, is involved in the pathogenesis of SLE (Berthelot et al., 2017; Kaufman et al., 2017; Pan et al., 2020). In addition to this mechanism, Th1, Th2, and Th17 cell dysfunction have been related to the occurrence and development of the disease. Under normal circumstances, Th1 and Th2 cells maintain an immune balance. However, the imbalance between these cells contributes to the pathogenesis of the disease. As for Th17 cells, IL-17 produced by these cells, associated with B-cell growth factor, positively regulates the differentiation and survival of B cells, stimulating humoral immunity to produce antibodies. Thus, SLE is characterized by intense production of autoantibodies, deposition of antigen–antibody complex and activation of the complement system in tissues, leading to the accumulation of self-reactive monocytes, neutrophils, and lymphocytes (Tsokos et al., 2016; Muhammad Yusoff et al., 2020; Pan et al., 2020).

The failure in immunological tolerance characteristic of SLE can be also promoted by dysbiosis or aberrant intestinal immunity (Jiao et al., 2020). Despite differences in dysbiosis patterns in the disease, studies have reported a reduction in the Firmicutes/Bacteroidetes ratio compared to healthy controls (Hevia et al., 2014; Rodríguez-Carrio et al., 2017; van der Meulen et al., 2019). As an example, in a Chinese population, in fecal samples from patients with SLE, a decrease in bacterial richness, a reduction in the Firmicutes/Bacteroidetes ratio and an increase in the relative abundance of *Bacteroides* was identified (Wei et al., 2019). In addition, an abundance of other genera has been demonstrated in individuals with SLE: *Rhodococcus*, *Eggerthella*, *Klebsiella*, *Prevotella*, *Eubacterium*, and *Flavonifractor* (He et al., 2016; Xu et al., 2019).

Dysbiosis may also be associated with the activity or remission phase of SLE, since affected individuals seem to exhibit characteristic patterns of dysbiosis in the intestinal microbiota in parallel with disease activity. Lupus activity was positively associated with the genera *Streptococcus*, *Campylobacter* and *Veillonella* and the species *S. anginosus* and *V. dispar*, while the genus *Bifidobacterium* was negatively correlated with disease activity (Li Y. et al., 2019). *Streptococcus* and *Veillonella* genera appear to have pro-inflammatory effects. *Streptococcus* combined with *Veillonella* obtained from the human intestine inhibited the production of IL12p70 and increased the response of TNF-α, IL-8, IL-6, and IL-10 (Figure 3; Van Den Bogert et al., 2016). Furthermore, through molecular mimicry, some *Streptococcus* species induce the activation of B cells and specific CD4+ T cells through antigen presentation (Blank et al., 2007). Therefore, these genera can interfere with the mucosal immune system and induce cross-reaction with host tissue, potentially being involved in enhancing the host’s immune response in SLE (Wang et al., 2022).

### 4.4. The gut microbiota and inflammatory skin diseases

There is increasing evidence that gut health exerts profound effects upon non-gastrointestinal diseases, including those of the skin (Searle et al., 2020). Intestine and skin are immunological barriers and constitute the environment for physiological microbiota (Polkowska-Pruszyńska et al., 2020). The concept of **gut–skin axis** has been implicated in the pathogenesis of many chronic inflammatory diseases. It suggests that the gastrointestinal system directly affects the skin homeostasis and allostasis through interactions between the immune, metabolic, and nervous systems (Wang and Chi, 2021). Gut dysbiosis has been implicated in many dermatologic conditions.

Intestinal microbiota dysbiosis has been shown in **psoriatic patients** and it correlates to the severity and status of the disease (Huang et al., 2019; Buhaș et al., 2022). Moreover, psoriatic patients showed less diversity in gut microbiota when compared to controls (Schade et al., 2022). It was hypothesized that the differential plenty of bacteria may be the reason for the gut dysbiosis in psoriasis instead of the number of bacterial species (Thye et al., 2022). A link between gut dysbiosis and butanoate metabolism and butyrate production has also been proposed, since it has been implicated in the regulation of various inflammatory factors, including TNF-α, IL-10, and Il-1𝛽 (Buhaș et al., 2022). It has been hypothesized that the presence of *Escherichia coli* could be related to psoriasis, since it was increased in intestinal flora of psoriatic patients. *E. coli* is known to be responsible for the production of TNF-α and other proinflammatory cytokines and also have been related to the etiology of IBD (discussed above), which is known to be related to psoriasis (Wen et al., 2023). Although the immunological and inflammatory responses in psoriatic patients seem to be affected by intestinal dysbiosis, the composition of the microbiota profile still needs more investigation since the results are heterogeneous (Buhaș et al., 2022).

The relationship between **atopic dermatitis** and gut microbiota was also studied. Various observational studies showed different results regarding the diversity and the composition of the gut microbiota in atopic dermatitis patients (Widhiati et al., 2021). Lower intestinal bacterial diversity has been associated with an increased risk of atopic disease (Polkowska-Pruszyńska et al., 2020). This dysbiosis results in a reduction of short-chain fatty acids production, like acetate, propionate, and butyrate. They are known to be potent anti-inflammatory in many diseases, including atopic dermatitis, through inhibition of Th2 and activation of regulatory T cells (Alam et al., 2022). These changes can cause a disruption in the integrity of the gut epithelial barrier, leading to an increased intestinal permeability and favoring toxins and gut microorganisms to penetrate the body circulation and contribute to skin inflammation. When these reach the skin, a strong Th2 reaction may be induced, causing further tissue damage (Moniaga et al., 2022). The use of probiotics was also studied, and some results point to an improvement on the severity of the atopic dermatitis (Petersen et al., 2019). Its role is based on their ability to balance the intestinal microbiota, protecting the gut barrier function, and decreasing the production of the pro-inflammatory cytokines IL-4, IL-5, IL-13, and TNF-α, which are closely related to atopic dermatites (Fang et al., 2021).

Microbial diversity is significantly decreased in **acne** patients when compared to controls(Deng et al., 2018). A decrease in *Lactobacillus*, *Bifidobacterium*, *Butyricicoccus*, *Coprobacillus*, and *Allobaculum* was found in patients with acne (Yan et al., 2018). *Lactobacillus* and *Bifidobacterium* are probiotic genera that balance the intestinal microbiota and also strengthen the intestinal barrier (Lee et al., 2019). Furthermore, the influence of dietary habits in acne supports the existence of gut-skin axis (Polkowska-Pruszyńska et al., 2020). Similar results were seen in **rosacea** patients, which present with similar quantity of bacteria, but a reduced richness on the composition (Chen et al., 2021)*. Acidaminococcus*, *Megasphaera* e *Lactobacillales* were genus more prevalent in rosacea patients, while *Peptococcaceae*, *Methanobrevibacter*, *Slackia*, *Coprobacillus*, *Citrobacter* e *Desulfovibrio* were reduced when compared to controls (Nam et al., 2018). Another study found increased abundance of *Rhabdochlamydia*, *Bifidobacterium*, *Sarcina*, *CF231*, *Ruminococcus* in rosacea patients and reduced quantity of *Lactobacillus*, *Roseburia*, *Megasphaerae*, *Acidaminococcus*, *Hemophilus*, *Citrobacter* and *Clostridium* (Chen et al., 2021).

In patients with **hidradenitis suppurativa** (HS), a reduction diversity was observed when compared to controls (McCarthy et al., 2022). One of the greatest differences were high degrees of *Ruminococcus gnavus* and *Clostridium ramosum*, which have already been related to Crohn’s disease (McCarthy et al., 2022). Different compositions in intestinal microbiota between HS patients and controls have also been demonstrated, with lower abundance of Firmicutes phyla (Kam et al., 2021). However, that was a pilot study and further investigation is still needed to corroborate these results. On the other hand, no differences in diversity were observed in another recent study, although there were some bacterial features differences (Lam et al., 2021). One interesting finding was the presence of *Robinsoniella* in 59% of HS patients and in none of the healthy controls (Lam et al., 2021). The gut dysbiosis described in these studies leads to an increased production of inflammatory cytokines such as TNF-α, IL-1β, and IL-6 by the intestinal epithelia which is followed by an increase in circulating inflammatory cytokines namely IFN-γ, and TNF-α. These cytokines end in an inflammatory process in the skin that involves MMP expression and are directly related to HS lesion formation (Molnar et al., 2020). It is also known that as HS, other diseases like psoriasis and IBD run with increased IL-17, and also present with gut microbiota alterations (Matusiak et al., 2017).

In **alopecia areata** (AA), two studies failed to demonstrate differences in diversity between patients and controls (Moreno-Arrones et al., 2020; Lu et al., 2021). However, at the genus level, abundance of *Blautia*, *Pseudomonas*, *Collinsella*, *Megasphaera*, and *Dorea* was found in AA patients (Lu et al., 2021). Other study found an elevated presence of *Holdemania filiformis*, Lachnospiraceae, Erysipelotrichaceae, *Parabacteroides johnsonii*, *Bacteroides eggerthii*, *Clostridiales vadin* BB60 group, *Eggerthellaceae* and *Parabacteroides distasonis*, while in controls, *Phascolarctobacterium succinatutens*, Clostridiales family XIII, *Dorea longicatena*, *Phocea massiliensis*, *Turicibacter sanguinis, Streptococcus thermophilus* and *Flavonifractor plautii* in patients with AA were notably more abundant (Moreno-Arrones et al., 2020). Moreover, improvement of AA symptoms was reported after fecal microbiota transplantation, reinforcing the association between intestinal microbiome composition and AA pathogenesis (Rebello et al., 2017; Xie et al., 2019). It is hypothesized that the gut microbiota could also interfere with **wound healing** by interfering with healing factors like tissue oxygenation levels, blood pressure, inflammation, and the immune system (Patel et al., 2022). Nevertheless, little is known about the intestinal microbiota composition in patients with chronic ulcers.

The impact of the gut microbiota is being studied in several other skin conditions, like **vitiligo**, **lichen sclerosus**, **seborrheic dermatitis,** and **skin cancer**, including the response to immunotherapy, like in **cutaneous melanoma** (Bzioueche et al., 2021; Chattopadhyay et al., 2021; Spencer et al., 2021). Although the relationship between the gut microbiota and these diseases is already established in most of them, there is still a gap to be filled in order to better understand its impact and mechanisms. The knowledge of such influence could shed some light on potential therapeutic allies, like probiotics, diet, and even fecal microbiota transplantation.

## 5. The gut microbiota and viral infections

As with each infection, each pathogen can induce a different immune response, as the process of activating these responses takes place. In a viral infection, the main cells involved in the process of fighting the virus are cytotoxic cells, which may be linked either to innate immunity (NK) or to adaptive immunity (CD8+), always with the fundamental antiviral action of the cytokine IFN-γ (Mazzoni et al., 2020).

It is well known that a healthy commensal microbiota is critical to protecting the host against a several of infections, either by direct elimination or by indirect suppression, inside or outside the intestine (Rothschild et al., 2018). The mucosal epithelium is the main entry route for many pathogens, which can cause an important dysbiosis by affecting the intestinal mucosal barrier (Rigo-Adrover et al., 2018). During viral infection in mucosal tissue, viruses may encounter the host’s commensal microbiota. Depending on the profile of this microbiota, it is possible that it is beneficial to the host, defending it from infections, as well as it is possible that it creates an environment conducive to viral infection (Table 2; Pfeiffer and Virgin, 2016; Schuijt et al., 2016).

**Table 2:** Viral diseases and alteration of the gut microbiota composition.

Disease	Species	Increase microbiota species	Depletion microbiota species
HBV	Human^1^, Mouse^2^	Genus: *Enterococcus*, Family: *Enterobacteriaceae* (Lu et al., 2011)^1^, genus *Faecalibacterium* and *Gemella* (Wang et al., 2017) ^1^	Genus: *Bacteroides* (Sender et al., 2016; Wang et al., 2017)^1^
			Species: *Bifidobacteria*, and *Lactobacilli* (Cosseau et al., 2008; Lu et al., 2011; Xu et al., 2012; Kakiyama et al., 2013; Aly et al., 2016; Wang et al., 2017; Inoue et al., 2018; Sultan et al., 2021b)^1^
HCV	Human^1^, Mouse^2^	Genus: *Prevotella*, *Succinivibrio*, *Catenibacterium*, *Megasphaera*; and family *Ruminococcacea* (Sultan et al., 2021b)^1^	Genus: *Bacterioides*, *Dialister*, *Bilophila*, *Streptococcus*, *Parabacterioides*; and families of *Enterobacteriaceae*, *Erysipelotrichaceae* and *Rikenellaceae* (Sultan et al., 2021b)^1^
			Phylum *Firmicutes*, Family *Ruminococcaceae* and *Lachnospiraceae* (Cosseau et al., 2008; Lu et al., 2011; Xu et al., 2012; Kakiyama et al., 2013; Aly et al., 2016; Wang et al., 2017; Inoue et al., 2018; Sultan et al., 2021b)^1^
		Family *Enterobacteriaceae*, Genus *Bacterioides* (Cosseau et al., 2008; Ponziani et al., 2018)^1^	
		Phylum *Proteobactérias*; Genus *Veillonella*, *Prevotella*, *Faecalibacterium*, *Acinetobacter*; *Streptococcus viridans*, *Streptococcus salivarius*; Families *Staphylococcaceae*, *Enterococcaceae*, *Veillonellaceae*, *Phascolarctobacterium* (Inoue et al., 2018; Ponziani et al., 2018)^1^	
		Family *Streptococcaceae* and *Lactobacillaceae* (Kakiyama et al., 2013; Tuomisto et al., 2014; Chen et al., 2016; Inoue et al., 2018)^1^	
COVID-19	Human^1^, Mouse^2^	Genus: *Streptococcus*, *Rothia*, *Veillonella* and *Actinomyces* (Gu et al., 2020)^1^	Genus: *Agathobacter*, *Fusicatenibacter*, *Roseburia*, family *Ruminococcaceae* (Gu et al., 2020)^1^
			Species: *Faecalibacterium prausnitzii* and *Eubacterium* rectale (Yeoh et al., 2021)^1^

^1^Human model. ^2^Mouse model. HBV, hepatitis type B virus; HCV, hepatitis C virus.

The commensal microbiota may help to promote viral infection by, for instance, facilitating viral gene recombination, thus allowing an increase in viral infectious capacity (Combe et al., 2015). The microbiota also may influence viral infection through other indirect mechanisms, such as stimulating the creation of immunoregulated environments through the production of IL-10 by Treg cells and the inhibition of cytokines such as IFN-γ and TNF-α, which disrupts the immune system’s ability to act properly to fight the viral infection (Basic et al., 2014; Robinson et al., 2014; Zhao and Elson, 2018).

On the other hand, intestinal microbiota is fundamental for the maturation of the immunological system and can also cooperate with it to prevent and fight infections. For instance, commensal populations can induce the immune system to produce antiviral products, such as IFN (Yitbarek et al., 2018). Among the different viral infections positively and negatively affected by the intestinal microbiota, and which are capable of also altering it, viral hepatitis (mainly HBV and HCV) and SARS-CoV-2 have been highlighted in the literature and will be discussed in further detail.

### 5.1. The gut microbiota and viral hepatitis

Viral hepatitis occurs due to infections of hepatitis A B, C, D, and E viruses, which are considered a public health issue, mainly in low and middle-income countries. Hepatitis type B virus (HBV) and Hepatitis C virus (HCV) are considered the most important etiological agents of hepatitis, whose infection can result in serious liver problems, including liver cirrhosis (LC), hepatocellular carcinoma (HCC) and liver failure. These clinical conditions can usually progress slowly and silently through various clinical stages as long these liver viruses have ways of preventing their detection by the host’s immune system, a characteristic called viral escape (Visvanathan et al., 2007; Lemon et al., 2018; Yang et al., 2018). Hepatitis A and E viruses, on the other hand, cause acute infection that can resolve independently of any intervention, unless the infected individuals are in an immunocompromised condition (Lemon et al., 2018).

Dysbiosis of the intestinal microbiota can be exploited by viral hepatitis as an escape mechanism of the immune system (Inoue et al., 2018; Li et al., 2018). Sender et al. (2016) and Wang et al. (2017) showed that the level of *Bacteroides* was lower in patients with hepatitis B compared to healthy people. Lu et al. (2011) suggested that cirrhosis could impact the dysbiosis process, leading to a worsening of the patient’s clinical condition. The intestinal microbiota also can be greatly affected during the different stages of HCV infection. During the asymptomatic phase, an increase in bacteria of the genera *Prevotella*, *Succinivibrio*, *Catenibacterium*, *Megasphaera* and from the *Ruminococcacea* family has been observed, as well as a reduction in bacteria from the genera *Bacteroides, Dialister, Bilophila, Streptococcus, Parabacterioides,* in addition to the following bacterial families: Enterobacteriaceae, Erysipelotrichaceae and Rikenellaceae (Sultan et al., 2021b). Studies have indicated evidence of dysbiosis since the onset of HCV infection, such as increased concentration of bacteria from the Enterobacteriaceae family and bacteria from the genus *Bacteroides* (Cosseau et al., 2008; Ponziani et al., 2018). Different studies have suggested HCV infection-related dysbiosis, can be intensified by the increased presence of bacteria from the phylum Proteobacteria Firmicutes and Bacteroidetes (Inoue et al., 2018; Ponziani et al., 2018). However, in dysbiosis related to chronic HBV infection, changes occur in the concentration of bacteria of the *Enterococcus* genus and the Enterobacteriaceae family, which may be increased (Lu et al., 2011; Wang et al., 2020).

In chronic HCV infection, bacteria from the Firmicutes phylum and Ruminococcaceae and Lachnospiracea families may occur, while in chronic HBV infection, Bifidobacteria, from the genus *Bifidobacterium,* and the intestinal *Lactobacilli*, from the genus *Lactobacillus*, seem to be less present due to dysbiosis process in chronic infection. On the other hand, in the development of cirrhosis (due to HCV infection), there may be an increase in the proliferation of bacteria from the genera *Enterobacteriaceae*, *Staphylococcaceae*, *Veillonellaceae* and *Bacteroides*, in addition to the phylum Proteobacteria (Cosseau et al., 2008; Xu et al., 2012; Kakiyama et al., 2013; Aly et al., 2016; Wang et al., 2017; Inoue et al., 2018; Sultan et al., 2021b).

According to Wang et al. (2017), the dysbiosis observed in chronic HBV infection is similar to that found in cirrhosis, with an increase in bacteria from the *Enterococcus*, *Faecalibacterium* and *Gemella* genera, and from the Enterobacteriaceae family, in addition to a decrease in *Bifidobacteria* and *Lactobacilli* in the intestinal microbiota. However, in the evolution to Hepatocellular Carcinoma, the dysbiosis caused by HCV seems to be distinct from the dysbiosis found in cirrhotic patients and chronic patients, as only the species *Streptococcus salivarius* and the families Streptococcaceae, Lactobacillaceae and Enterobacteriaceae seem to be elevated, while only Ruminococcaceae and Lachnospiraceae would have a reduction (Kakiyama et al., 2013; Tuomisto et al., 2014; Chen et al., 2016; Sanduzzi Zamparelli et al., 2017; Inoue et al., 2018).

The dysbiosis process may be accompanied by liver inflammation, allowing the evolution to cirrhosis and hepatocellular carcinoma due to action of pro-inflammatory cytokines with a Th1/Th17 profile (Rigo-Adrover et al., 2018). Dysbiosis in patients with cirrhosis and hepatocellular carcinoma can strongly affect the permeability of the mucosal tissue, allowing the induction of the innate immune system of the liver. Thus, it is possible that the liver damage found in these patients is not only due to the antigen-specific cellular immune response in response to viral action, but also due to pathogen-associated molecular patterns (PAMPs), which also can trigger an innate immune response and, eventually, tissue damage. For example, patients chronically infected with HBV may have a reduction in the presence of *Lactobacilli* and *Bifidobacteria* in their intestinal microbiota. Both bacterial families are rich in unmethylated CpG DNA, which directly triggers the CpG DNA-TLR9 pathway and the immune response to the liver virus. Unmethylated CpG DNA are recognized as PAMPs by TLR9, which is expressed in several mononuclear cells, stimulating the innate and adaptive immune response (Wu et al., 2011; Xu et al., 2012; Ries et al., 2013).

### 5.2. The gut microbiota and SARS-CoV-2 infection

Infection by SARS-COV-2, which causes the pathology called Covid-19, is still under intensive investigation due to its unique characteristics. In addition to COVID-19 being a respiratory viral infection, different clinical pictures, and a major feature of the infection’s aggravation is the cytokine storm and the development of an intense inflammatory response (Vabret et al., 2020).

The pathophysiology of this infection is directly related to this intense inflammatory response. Thus, the severity of the disease is often not only related to the viral infection, but also to the exacerbated immune response of the host. Patients with severe COVID-19 exhibit elevated levels of inflammatory markers such as IL-6, IL-8, C-Reactive Protein (CRP) and lactate dehydrogenase (LDH). SARS-CoV-2 utilize the angiotensin-converting enzyme receptor 2 (ACE2) to penetrate the host’s target cell. ACE2 is highly expressed not only in the respiratory tract but also in various other tissues, including the gastrointestinal tract. This important aspect of infection is further evidenced by the fact that ACE2 is important in controlling inflammation and the intestinal microbiota (Lamers et al., 2020; Vabret et al., 2020; Zuo et al., 2020). After virus entry, various inflammatory signaling pathways are activated within cells and inflammatory products are released. Among these products, type I Interferons (IFN-I) are essential in the first line of defense, creating an antiviral environment that makes it difficult for the perpetuation of the virus. However, SARS-COV-2 has the ability to evade the immune system by inhibiting the production of IFN-I (Lamers et al., 2020; Tay et al., 2020; Vabret et al., 2020; Zuo et al., 2020; Yeoh et al., 2021).

SARS-Cov-2 has already been detected in fecal samples and there is evidence that this virus replicates in enterocytes, which could promote alterations in the intestinal microbiota in patients who developed COVID-19 (Lamers et al., 2020). Zuo et al. (2020) identified persistent changes in the fecal microbiome of patients with COVID-19 during their hospital stay, compared to controls. These changes in the fecal microbiota were associated with fecal levels of virus and gravity of COVID-19. Furthermore, bacterial species of Bacteroidetes appeared to be negatively correlated with the severity of COVID-19. Species of the genus *Bacteroides,* such as *B. dorei*, were inversely correlated to the fecal viral load of SARS-COV-2, and it is possible that *B. dorei* induces suppression of ACE2 expression (Zuo et al., 2020). On the other hand, Agathobacter, Fusicatenibacter, Roseburia, and Ruminococcaceae were less present in COVID-19 patients, being negatively correlated with CRP, procalcitonin and D-dimer levels. A reduction in the presence of bacterial with immunomodulatory activity, such as *Eubacterium rectale*, *Faecalibacterium prausnitzii*, and *Bifidobacterium* was observed. Conversely, CRP and D-dimer levels were positively correlated with the increased expression of *Streptococcus*, *Rothia*, *Veillonella* and *Actinomyces* bacteria (Gu et al., 2020). In summary, the composition of the intestinal microbiota in patients with COVID-19 has been correlated to the severity of the disease. Dysbiosis may remain present in the patient’s intestinal microbiota even after recovery from SARS-COV-2. The alteration of these bacterial groups was also associated with the elevation of the cytokines TNF-α, CXCL10 (C-X-C Motif Chemokine Ligand 10), CCL2 (C-C motif chemokine ligand 2) and IL-10. So, it is possible that this dysbiosis is related to the more severe version of the pathology of COVID-19, where there is an intense production of proinflammatory cytokines (Yeoh et al., 2021).

## 6. The gut microbiota and mycobacteria infections

### 6.1. Tuberculosis

Tuberculosis (TB) is an infectious disease caused by the alcohol-acid-resistant bacillus *Mycobacterium tuberculosis* and is considered one of the main neglected diseases in the world (Eribo et al., 2020). It is a highly transmissible disease spread by aerosol droplets containing bacilli, usually during sneezing or coughing (Eribo et al., 2020; Global Tuberculosis Report, 2020). It is believed that in most individuals the infection results in clinically asymptomatic latent tuberculosis infection (Eribo et al., 2020; Mori et al., 2021). The bacillus predominantly infects the lungs, causing pulmonary tuberculosis. However, it can also invade extrapulmonary organs such as lymph nodes, bones, and meninges (Ko et al., 2000; Hu et al., 2019). Although 90%–95% of individuals infected with *M. tuberculosis* remain protected throughout their lifetime, 5%–10% of people develop active tuberculosis (Nadeem et al., 2020). Immune, host genetic and environmental predisposing factors, such as HIV infection and diabetes, have been associated with the disease (Eribo et al., 2020; Mori et al., 2021). During active tuberculosis, symptoms include cough, fever, weight loss and hemoptysis (Lyon and Rossman, 2016).

The gut microbiota has been reported as a host factor that may be associated with tuberculosis (Hudrisier et al., 2018; Khan et al., 2019). Studies have shown remarkable differences between the gut microbiota of TB patients and healthy controls (Luo et al., 2017; Hu et al., 2018). Hu et al. (2018) reported a decrease in microbiome diversity, mainly associated with changes in the relative abundance of Bacteroides in the gut microbiota of Chinese patients with TB. In another study, an important decrease in the number and diversity of the microbiota was observed, with a remarkable reduction in SFCA-producing bacteria such as *Roseburia inulinivorans*, *Bifidobacterium adolescentis*, and *Akkermansia muciniphila* (Hu et al., 2019).

Luo et al. (2017) divided patients analyzed by them according to time of diagnosis and treatment time into new tuberculosis patients and recurrent tuberculosis patients. New tuberculosis patients showed an increase in Actinobacteria and Proteobacteria, while recurrent patients showed a reduction in Bacteroidetes, containing several beneficial commensal bacteria in fecal samples. The phylum Proteobacteria contains several gram-negative bacteria and opportunistic pathogenic species (Luo et al., 2017). The lipopolysaccharide (LPS) component of the cell wall of these bacteria can trigger the activation of pro-inflammatory macrophages (M1) and other innate immune cells (Sommer and Bäckhed, 2013; Mori et al., 2021). M1 macrophages are characterized by high antigen presentation and expression of IL-12, IL-23 and TNF-α (X). Therefore, this group of bacteria can induce an inflammatory response locally and at distant sites if the epithelial barrier is disturbed (Sommer and Bäckhed, 2013; Mori et al., 2021). Since any damage to the intestinal barrier can cause microbial translocation into the blood and produce a sustained inflammatory response, it might also impact lung disease (Ma P. J. et al., 2022). In the same research, *Prevotella* and *Lachnospira* were considerably reduced in new and recurrent tuberculosis patients compared to healthy subjects (Table 3; Luo et al., 2017; Li W. et al., 2019; Liu et al., 2021). Furthermore, *Prevotella* was positively correlated with the number of peripheral CD4+ cells in NTB and negatively correlated with RTB (Luo et al., 2017). Taken together, these data suggest that specific intestinal microorganisms may modulate the host immune system and be related to patient prognosis and outcome, especially in cases of impaired intestinal barrier (Luo et al., 2017; Li W. et al., 2019).

**Table 3:** Tuberculosis and alteration of the gut microbiota composition.

Disease	Species	Increase microbiota species	Depletion microbiota species
TB	Human^1^, Mouse^2^		Genus *Bacteroides* (Hu et al., 2019)^1^
			Species: *Roseburia inulinivorans*, *Bifidobacterium adolescentis* and *Akkermansia muciniphila* (Hu et al., 2019)^1^
NTB	Human^1^, Mouse^2^	Phylum: *Actinobacteria* and *Proteobacteria* (Luo et al., 2017; Li W. et al., 2019)^1^	Genus *Prevotella* and family *Lachnospira* (Luo et al., 2017; Li W. et al., 2019)^1^
RTB	Human^1^, Mouse^2^		Phylum *Bacteroidetes*, genus *Prevotella* and family *Lachnospira* (Luo et al., 2017; Li W. et al., 2019)^1^

TB, tuberculosis; NTB, new tuberculosis patients; RTB, recurrent tuberculosis patients.

### 6.2. Leprosy

Leprosy is a chronic granulomatous mycobacteriosis with high infectivity and low pathogenicity, and, like tuberculosis, is considered one of the main neglected diseases. The disease is caused by *Mycobacterium leprae* and occurs in a variety of clinical forms that depend on the immune status of the host (Costa et al., 2018; Pinheiro et al., 2018). The disease especially affects the skin and peripheral nerves, but it can also affect the eyes, upper respiratory tract mucosa, bones, and testicles (Desikan and Iyer, 1972; Pinheiro et al., 2018). Classically, it is characterized with a Th1/Th2 paradigm, presenting a cytokine profile that varies according to the type of Th response. However, studies have also shown differences across the disease spectrum for Th9, Th17, Th25 and Treg lymphocytes (de Sousa et al., 2017; Froes et al., 2022).

Leprosy has been associated with dysbiosis of the skin microbiota. Atypical human skin taxa were identified in leprosy lesions, with the genera *Burkholderia*, *Pseudomonas* and *Bacillus* being overrepresented (Silva et al., 2018), while the *Staphylococcus* genus, which is inhabitant and abundant in healthy people skin, was underrepresented in these lesions when compared to healthy controls (Silva et al., 2015; Bayal et al., 2019). A study evaluated the constitution of the skin microbiome in lepromatous skin lesions (and matched adjacent uninjured areas) sampled from a cohort of Brazilian patients. The researchers found in both samples from infected leprosy patients (injured and uninjured tissue) less diversity compared to the skin of healthy individuals (Silva et al., 2018). This lower diversity could be imputed to the impact of the microorganism itself or to a systemic change resulting from the ongoing treatment regimen (Bayal et al., 2019).

Two main types of reactions can occur in leprosy patients, the reverse reaction and erythema nodosum leprosum (ENL). The reverse reaction is an acute inflammatory episode in the skin and nerves characterized by an accentuated of the cellular immune response against *M. leprae.* ENL is a systemic inflammatory process characterized by an increase in the levels of pro-inflammatory cytokines, such as TNF-alpha, IL-6, and IL-1B, derived from Th17 lymphocytes (Costa et al., 2018; Froes et al., 2022). Taking into account that the gut microbiota influences the homeostasis of various populations of T cells in the gut, including Tregs, Th1 and Th17 (Gaboriau-Routhiau et al., 2009), directing the pattern of local and systemic immune response (Schirmer et al., 2016; Thursby and Juge, 2017), it is plausible to assume that the host’s gut microbiota may be associated to the variety of clinical responses present in leprosy and to the inflammatory state in leprosy reactions. However, so far there are no studies evaluating the role of the intestinal microbiota in leprosy and in the development of leprosy reactions.

## 7. Discussion and conclusion

The microorganisms present in the human intestine play a key role in the process of development, maturation, and maintenance of the action of the body’s defense cells. They play a key role in host homeostasis under basal conditions. In addition to the competition with different pathogens for different niches, they induce protective responses through the modulation of inflammatory responses with the production of cytokines. Allied to the stimulation of protection against pathogens, the tolerance mechanisms promoted by a healthy intestinal microbiota, which involve the reduction of physiological impairment originated by interplay with microorganisms, constitute a strategy of equal significance for the conservation of the host’s health that enable the co-evolution of microorganism-host interactions. Despite this, small disparity in this interplay can have negative health implications and dysbiosis can lead to increased susceptibility to infections, as well as an imbalance in the host defense system, resulting in multifactorial inflammatory diseases.

Evidence of the ways in which microbiota influences host physiology suggests that interplay between microbiota and cytokine pathways may be critical to sustaining host inflammation both in the gut and at extraintestinal sites. As an example, IL-17 can modulate, and be modulated by the composition of the microbiota (Valeri and Raffatellu, 2016; Douzandeh-Mobarrez and Kariminik, 2019). Mice with microbiota depleted by antibiotic have reduced levels of IL-17 in the lamina propria of the small intestine (Hill et al., 2010) and mice with RA treated with antibiotics show reduced Th17 cells and IL-17 levels in the gut, reducing the consequences of arthritis (Rogier et al., 2017). These data exhibited the relevance of the intestinal microbiota in modulating the production of IL-17 and associated the overexpression of this cytokine to the development of RA (Eyerich et al., 2017).

In addition, in IBD, RA, COVID-19 and tuberculosis there is a reduction in the abundance of butyrate-producing bacteria (Sokol et al., 2009; Chen et al., 2016; Hu et al., 2019; Yeoh et al., 2021). Especially *Faecalibacterium* species, which are diminished in three of these pathologies (Sokol et al., 2009; Chen et al., 2016; Yeoh et al., 2021). As mentioned earlier, butyrate is an SCFA that plays a fundamental role in conserving the integrity of the intestinal mucosa and in the balance between Treg and Th17 cells (Kim et al., 2016; Zhou et al., 2018). Thus, the change in the abundance of this bacterium could be related to a greater exposure of the host’s immune cells to intestinal bacteria, generating excessive activation of the immune system and an imbalance between T cells. Therefore, resulting in the intense inflammatory response involved in these diseases, whether autoimmune or infectious (IBD, AR and COVID; Sokol et al., 2009; Chen et al., 2016; Yeoh et al., 2021). Taken together, these findings exemplify the importance and influence that the gut microbiota can exert on the host’s immune response.

The microbiota-host cytokine relationship is a dynamic and complex process, where several factors can have a major effect on inflammation. The cytokine interaction patterns of the microbiota are stimulus-specific, cytokine-specific and cytokine and stimulus-specific, and are therefore disease-specific. In addition to the influence of the gut microbiome, environmental and host factors (genetic and non-genetic) also have an impact on cytokine modulation. Based on the data reviewed here, we can suggest that the gut microbiota has an important relevance in the outcome of infectious and inflammatory diseases by directing Th cell responses and by producing proinflammatory cytokines. Inflammatory diseases in which the intestinal microbiota has not been investigated, such as leprosy and its reactions, which are characterized by an intense increase in pro-inflammatory cytokines, may be at least in part related to alterations in the gut microbiota. Mainly because patients undergoing treatment for leprosy, for example, receive antibiotic treatment for up to 12 months (Costa et al., 2018). Therefore, it could lead to significant changes in the intestinal microbial community, with possible consequences for the modulation of immune responses and the development of inflammatory reactions. However, further studies are needed to clarify whether dysbiosis is the cause or consequence of the pathologies studied here and other diseases with an inflammatory background. Seeking to understand which changes in the intestinal microbiota or metabolites influence the variability of human cytokine responses in immunological diseases.

## 8. Future perspectives

As reviewed in this article, the gut microbiota has been associated with many diseases by inducing immune responses in different inflammatory conditions. Here, we provide a comprehensive overview of some of the possible cytokine modulation mechanisms by the microbiota already reported. However, it is fundamental taking into account that most studies use sequencing and analysis of 16S rRNA to infer to role of the microbiota in the health-disease interface. In order to obtain an in-depth view of the role of intestinal bacteria in the host’s immune system, it is essential that a greater number of studies assess, in addition to the composition, the metabolic patterns of the intestinal microbiota through metabolomic analyses. As highlighted here, microbial metabolites can directly or indirectly influence the pathophysiological states of the host, developing both pro- and anti-inflammatory effects. The future challenge will be to understand microbiota-metabolite-host interactions at the molecular level and in its entirety. A better understanding of these interactions can open perspectives for understanding and of biological pathways, as well as for adjuvant treatments based on probiotics containing immunoregulatory bacteria, prebiotics, which influence the growth of beneficial bacteria populations, or even a simple intervention in the diet.

## Author contributions

MM-F and GM contributed to the conception of the study, searching the literature, creating graphical illustrations, and writing the manuscript. DC contributed searching the literature and designed the tables. JP and RB contributed writing the manuscript. PS and TV contributed correcting the manuscript. FV contributed devising the concept, writing, and correcting the manuscript. All authors contributed to the article and approved the submitted version.

## Funding

This work was supported by Instituto Nacional de Genética Médica Populacional (INAGEMP; grant nos. CNPq 573993/2008-4 and FAPERGS 17/2551.0000521-0), Fundo de Incentivo à Pesquisa e Eventos (FIPE) of the Hospital de Clínicas de Porto Alegre (HCPA; grant no. 2019-0155), Coordenação Brasileira de Aperfeiçoamento de Pessoal de Nível Superior (CAPES), and Fundação de Amparo à Pesquisa do Rio Grande do Sul (FAPERGS) (grant no. 19/2551-0001787-1).

## Conflict of interest

The authors declare that the research was conducted in the absence of any commercial or financial relationships that could be construed as a potential conflict of interest.

## Publisher’s note

All claims expressed in this article are solely those of the authors and do not necessarily represent those of their affiliated organizations, or those of the publisher, the editors and the reviewers. Any product that may be evaluated in this article, or claim that may be made by its manufacturer, is not guaranteed or endorsed by the publisher.
